# Morphometric analysis of a triple negative breast cancer cell line in hydrogel and monolayer culture environments

**DOI:** 10.7717/peerj.4340

**Published:** 2018-02-16

**Authors:** Manasi P. Jogalekar, Elba E. Serrano

**Affiliations:** 1 Department of Biology, New Mexico State University, Las Cruces, NM, United States of America

**Keywords:** Autophagy, Confocal microscopy, Hydrogel, Triple negative breast cancer, Tissue engineering, Phase contrast microscopy, Ultrastructure, Monolayer culture, Tridimensional culture, Transmission electron microscopy

## Abstract

Triple negative breast cancer (TNBC) is a belligerent carcinoma that is unresponsive to targeted receptor therapies. Development of new treatment strategies would benefit from an expanded repertoire of *in vitro* cell culture systems, such as those that support tridimensional growth in the presence of hydrogel scaffolds. To this end, we established protocols for maintenance of the TNBC cell line HCC70 in monolayer culture and in a commercially available basement membrane matrix hydrogel. We evaluated the general morphology of cells grown in both conditions with light microscopy, and examined their subcellular organization using transmission electron microscopy (TEM). Phase contrast and confocal microscopy showed the prevalence of irregularly shaped flattened cells in monolayer cultures, while cells maintained in hydrogel organized into multi-layered spheroids. A quantitative ultrastructural analysis comparing cells from the two culture conditions revealed that cells that formed spheroids comprised a greater number of mitochondria, autophagic vacuoles and intercellular junctions than their monolayer counterparts, within the equivalent area of sampled tissue. These observations suggest that triple negative breast cancer cells in culture can alter their organelle content, as well as their morphology, in response to their microenvironment. Methods presented here may be useful for those who intend to image cell cultures with TEM, and for investigators who seek to implement diverse *in vitro* models in the search for therapeutic molecular targets for TNBC.

## Introduction

Triple negative breast cancer (TNBC) is an invasive cancer that spreads rapidly, weakens the body and can cause discomfort and pain. TNBC can manifest at all ages and is prevalent in women of African-American descent (Stead et al., 2009). Studies have unraveled molecular features of TNBC and this type of breast cancer is so named because TNBC cells lack receptors for estrogen, progesterone and human epidermal growth factor (Ovcaricek et al., 2011). Genetic factors can impact disease progression. For example, mutations in BRCA genes further increase the susceptibility of women to this disease (Meyer et al., 2012; Kuo et al., 2012; Atkinson et al., 2013). As a result, many current therapies are not effective treatments for TNBCs, leading to lower survival rate in patients (Anders, Zagar & Carey, 2013). Poor prognosis and a high recurrence have also been linked with TNBC resistance to chemotherapy and radiation therapy (Dent et al., 2007; Zhang et al., 2008; Atkinson et al., 2010; Boyle, 2012). Some reports suggest that an increase in cancer stem cells and autophagy may play a major role in imparting chemo- and radio-resistance to TNBC cells (Atkinson et al., 2013; O’Reilly et al., 2015). The mandate for new treatments against TNBC is spurring development of innovative approaches, such as the use of iron oxide nanoparticles and temperature-sensitive liposomes conjugated with therapeutic drugs (Guo et al., 2014; Ou et al., 2016).

*In vitro* cell culture models are widely used to study the pathology of cancer types, including TNBC (Kao et al., 2009; Grigoriadis et al., 2012). Monolayer culture environments typically cause the cells to grow in an apical-basal polarity with only one surface attached to the substrate, and this asymmetry alters cellular morphology and function from what is observed in the tissue of origin (Baker & Chen, 2012; Lovitt, Shelper & Avery, 2014). For these reasons, 2D culture models have been critiqued as suboptimal systems for predicting *in vivo* responses, in part because the microenvironment changes cell-to-cell communication and signaling from the native state (Jorgensen & Tyers, 2004). In contrast, three-dimensional (3D) culture systems provide a novel approach to assess the growth and behavior of cells (Petropolis et al., 2014; Warnock et al., 2014; Nath & Devi, 2016).

Previous studies conducted using human embryonic cells, hepatocytes, and melanoma cells have established that 3D microenvironments allow cells to grow in multiple layers by invading and penetrating the matrix scaffold and forming more natural intercellular junctions, thereby leading to better cell-to-cell communication and signaling (Jorgensen & Tyers, 2004; Ma et al., 2011; Baker & Chen, 2012; Leight et al., 2015). 3D cell culture systems typically incorporate scaffold materials, such as hydrogels, due to their similarity to *in vivo* conditions, especially to pathologies like cancer, as opposed to 2D models (Li, Fan & Houghton, 2007; Whiteside, 2008; Tibbitt & Anseth, 2009; Pontes Soares et al., 2012; Lovitt, Shelper & Avery, 2014; Xu et al., 2014). As a result, these 3D cultures may provide a powerful platform for anti-cancer drug screening (Herrmann et al., 2014; Xu, Farach-Carson & Jia, 2014; Zanoni et al., 2016; Cavo et al., 2016).

Determination of the similarity between cancer *in vitro* model systems, such as cells in hydrogel cultures as compared with the native tumor state, requires cellular and molecular analysis. In particular, because morphology is a prominent indicator of the invasive and metastatic state of cancer cells, morphological analysis plays a key role in the validation of experimental systems that aim to emulate the cancer tumor environment such as tumor explants, scaffold-based or scaffold-free spheroids, and tumor-on-a-chip (Nath & Devi, 2016). Previous studies using cancer cell lines have demonstrated that breast cancer cells display histopathological markers of invasion and metastasis such as tumor size, nuclear and histological grade, and axillary lymph node status (Emerman, Burwen & Pitelka, 1979; Weigelt, Geyer & Reis-Filho, 2010; Moosavi et al., 2014). Analysis of TNBC morphology in response to hydrogel environment has received minimal attention, especially using transmission electron microscopy (TEM) in conjunction with quantitative methods (O’Brien et al., 2013; Brand et al., 2014; Zhou et al., 2017). These observations prompted our interest in using TEM to quantify ultrastructural features of TNBC cells cultured in two conditions—monolayer and hydrogel.

In the present study, we established monolayer and 3D cell culture systems with the TNBC cell line, HCC70. Geltrex™, an extracellular basement membrane matrix, was chosen for our 3D cell culture experiments because it is derived from a natural material—Engelbreth-Holm-Swarm (EHS) tumors, and comprises essential proteins (e.g., laminin, collagen IV, entactin) and carbohydrates (e.g., the heparin sulfate proteoglycans). We compared cell growth in monolayer and hydrogel conditions through visualization of cell cultures using light and confocal microscopy. We conducted quantitative morphological analysis with TEM to evaluate the ultrastructural differences in cells grown in the two environments.

Our results demonstrated distinct morphologies of HCC70 cells, when cultured on the 2D rigid substratum and the 3D basement membrane matrix. In monolayer culture, the cells exhibited a characteristic phase-dark flat morphology and spread across the surface of a tissue culture slide. In contrast, the cells in hydrogel cultures grew in close proximity to one another and formed compact spheroidal structures. TEM highlighted the ultrastructural features enhanced in hydrogel cultures. Quantitative analysis of TEM images revealed that cells cultured in hydrogel comprised a higher number of mitochondria, autophagic vacuoles and intercellular junctions than cells from monolayer cultures. Taken together, these results suggest that cellular morphology and organelle abundance were influenced by the culture microenvironment. Our hydrogel culture methods may be useful for uncovering mechanisms of therapeutic resistance and for screening of therapeutic drugs to treat TNBC using novel methods such as nanodelivery of anti-cancer compounds (Guo et al., 2014; Ou et al., 2016). Moreover, the implementation of a variety of monolayer and hydrogel culture systems may be helpful for elucidating aspects of cancer pathology such as proliferation and cell–cell communication.

## Materials and Methods

### Cell culture and experimental samples

HCC70, a cell line derived from primary ductal carcinoma of an African-American female donor, was cultured in parallel in monolayer and in hydrogel scaffolds. HCC70 cells were cultured using biosafety level 2 (BSL2) aseptic technique without antibiotics. All cell culture reagents were either purchased sterile or filter-sterilized with 0.2 µm membrane filters. HCC70 cells (ATCC^®^ CRL2315^™^, Lot #58033362; ATCC, Manassas, VA, USA) were cultured as per the manufacturer’s instructions (ATCC, Manassas, VA, USA). Stock ampules (termed Passage 1) were prepared from the vendor’s vial when cells were received on dry ice in a frozen vial as previously described (Jogalekar, Cooper & Serrano, 2017). For each experiment, one Passage 1 stock ampule was seeded into two T25 flasks. Cells were cultured in RPMI-1640 media (ATCC, Lot #62285353; Manassas, VA, USA) and 10% fetal bovine serum (FBS; Lot #1025354; Gibco, Carlsbad, CA, USA) in an incubator maintained at 37 ^∘^C and 5% CO_2_. Once the cells reached 80% confluence, (∼48 h), they were trypsinized, titered, and seeded at a density of 4.5 ×10^4^ cells per cm^2^ in two, 4-well slides. Monolayer and hydrogel cultures were established on each slide with two chambers designated for each condition. One slide was designated for light microscopy (VWR, Cat #62407-294; Radnor, PA, USA) and the other was processed for TEM (Cat #62407-330; Nalge Nunc International, Penfield, NY, USA).

### Matrix preparation

Geltrex™ LDEV-Free Reduced Growth Factor Basement Membrane Matrix (Cat #A1413202; Invitrogen, Carlsbad, CA, USA) was allowed to thaw at 4 °C. The matrix was triturated gently with a pipette to prevent the formation of bubbles, prior to adding 100 µl of matrix per cm^2^ to the slides under sterile conditions. The Geltrex ^TM^ was allowed to solidify in 37 °C incubator for 30 min, before the cells were seeded on top of the matrix.

### Phase contrast microscopy

Prior to fixation for confocal microscopy, live cultures were imaged using 20 × objective of an inverted Nikon TE-2000 microscope equipped with a 2.8MP CoolSNAP MYO CCD camera (Photometrics) and Metavue image capture software (Molecular Devices).

### Sample preparation for confocal microscopy

Sample processing was carried out as described previously (Jogalekar, Cooper & Serrano, 2017). Live cultures were rinsed with 1 × Phosphate buffered saline, pH 7.4 (PBS, P3813; Sigma Aldrich, St. Louis, MO, USA) for 2 min and fixed for 10 min using 4% paraformaldehyde (PFA; CAS #30525-89-4; Electron Microscopy Sciences, Hatfield, PA, USA). The cells were washed twice with 1 × PBS for 2 min each and treated with 0.2% Triton X-100 (Cat #T8787; Sigma Aldrich, St. Louis, MO, USA) for 20 min. The cells were rinsed briefly twice with 1 × PBS, probed with 165 nM Alexa Fluor^®^ 488 phalloidin dye (Cat #A12379; Invitrogen, Carlsbad, CA, USA) in 1% bovine serum albumin (BSA; Cat #A2153; Sigma Aldrich, St. Louis, MO, USA), for 20 min in the dark. The cells were washed briefly twice with 1 × PBS and counterstained with 2 µg/ml Hoechst 33342 (Cat #H3570; Invitrogen, Carlsbad, CA, USA) in 1 × PBS, for 10 min in the dark. Equal amounts of 1% BSA in 1 × PBS was added to the auto-fluorescence controls. The cells were briefly rinsed twice with 1 × PBS and re-fixed in 4% PFA, before exposing them to SlowFade^®^ AntiFade kit (Cat #S2828; Invitrogen, Carlsbad, CA, USA). Coverslips were placed on samples and adhered to the slide with nail polish prior to imaging.

### Confocal microscopy

The digitized images of monolayer and 3D samples were captured using a TCS SP5 II confocal microscope system (Leica Microsystems Inc., Buffalo Grove, IL, USA) using a 40X objective (NA–0.70) and a pinhole of 1 Airy Unit. Alexa Fluor^®^ 488 phalloidin was excited using the 65 mW Argon laser line and images were captured with Alexa 488 emission settings. Hoechst 33342 was excited using a 50 mW 405 diode UV laser line and images were captured with DAPI emission settings. TIF image stacks were used for confocal projection images.

### Sample processing for TEM

Samples were processed for TEM as described previously (Jogalekar, Cooper & Serrano, 2017). The cells were fixed overnight at 4 ^∘^C (∼16 hr) with a fixative containing 2.5% glutaraldehyde (CAS #111-30-8; Electron Microscopy Sciences, Hatfield, PA, USA) and 0.1 M cacodylate buffer (#11650; Electron Microscopy Sciences, Hatfield, PA, USA) followed by washing twice with 0.1 M cacodylate buffer, 10 min each. The post-fixation was completed with 2% osmium tetroxide (CAS #20816-12-0; Electron Microscopy Sciences, Hatfield, PA, USA) / 0.1 M cacodylate buffer for 2 hr. The cells were rinsed in double distilled water (ddH_2_O) for 15 min and subjected to dehydration in a series of ethyl alcohols -50%, 70% and 95% (twice, 10 min each) and 100% (thrice, 10 min each). Cells were incubated in the transitional solvent containing a mixture of acetone: ethanol (2:3) thrice for 10 min each. Infiltration was carried out with 1:1 and 1:3 proportion of absolute ethanol: EMbed 812 resin (#14120; Electron Microscopy Sciences, Hatfield, PA, USA) for 30 min each, before incubating in pure resin for 15 min. Fresh resin was added to slides before they were cured for 48 hr in a 60 °C oven. The chambers were detached from the slide and immersed in liquid nitrogen to separate the resin blocks from the slide. The blocks were double-embedded in fresh resin and cured for 48 hr in a 60 °C oven before preparing ultra thin sections (∼70 nm) with a UC6 ultra-microtome (Leica Microsystems Inc., Buffalo Grove, IL, USA). Sections collected on Formvar carbon grids (Cat #FF100-Cu; Electron Microscopy Sciences, Hatfield, PA, USA) were stained with 2% uranyl acetate solution (#22400; Electron Microscopy Sciences, Hatfield, PA, USA) for 10 min, then rinsed with ddH_2_O, dried, and stored in grid boxes.

### Transmission electron microscopy

A transmission electron microscope, H-7650 (Hitachi High-Technologies, Pleasanton, CA, USA) and a CCD camera (AMT Corp., Woburn, MA, USA) were used to capture digital images of 2, 944 × 2, 944 pixel frame size acquired with an 80 kV accelerating voltage.

### Image analysis for TEM

**Figure 1:**
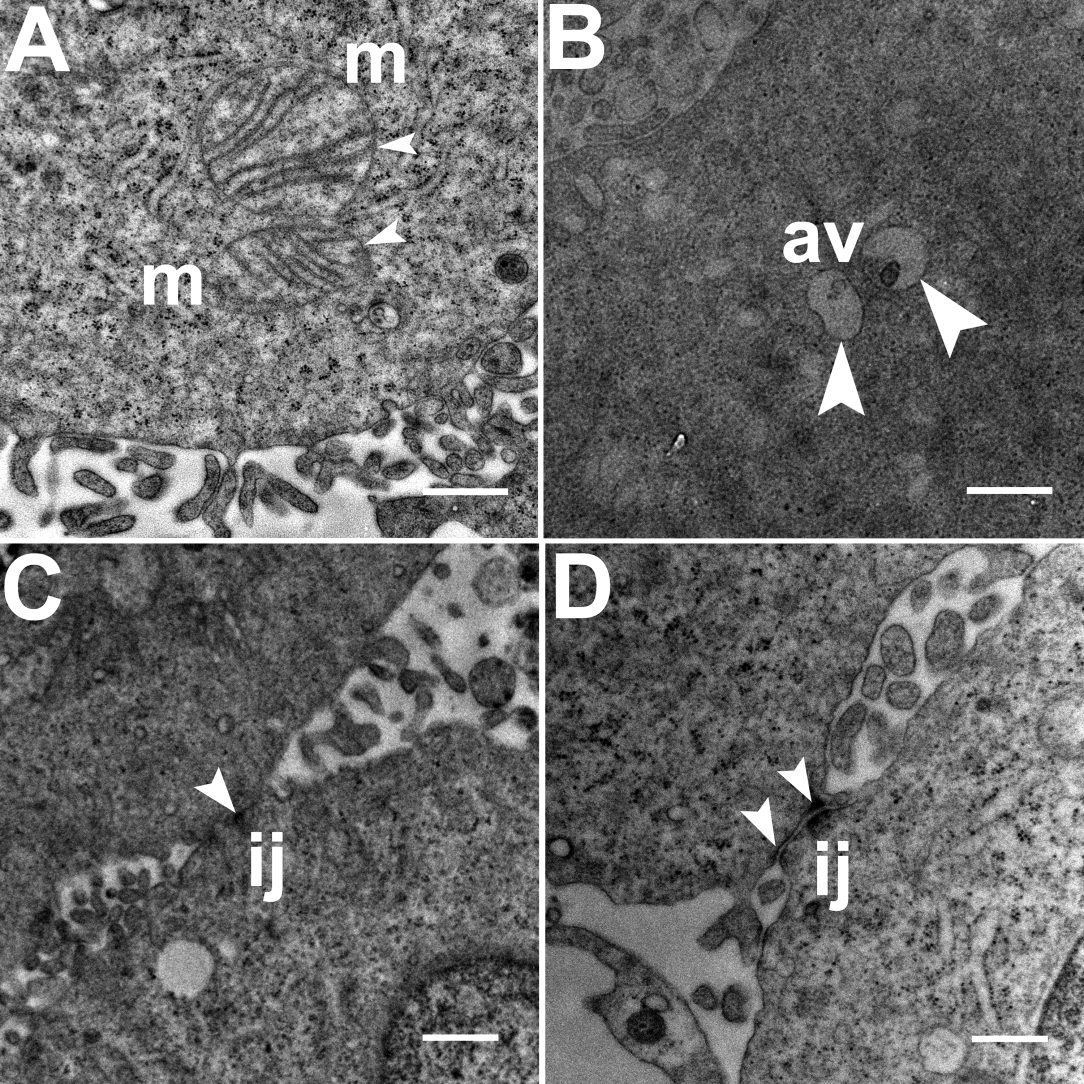
Exemplar TEM images of mitochondria, autophagic vacuoles and intercellular junctions. (A) The membrane enclosed structures containing filamentous cristae were identified as mitochondria (white arrowheads, ‘m’). Both round and cylindrical mitochondria were observed. (B) Membrane-enclosed structures comprising what appeared to be electron dense and partially digested cellular material, were identified as autophagic vacuoles (white arrowheads, ‘av’). (C, D) Intercellular junctions (white arrowheads, ‘ij’) were identified by the presence of electron-dense regions on the apposing membranes of adjacent cells. Scale bar = 500 nm.

Three grids were analyzed from each resin block and one image was captured for each grid, yielding a total of six images per chamber. The number of mitochondria, autophagic vacuoles and intercellular junctions was determined from all images at the same magnification (15, 000 ×, 80 kV) using the ‘count’ tool in Adobe Photoshop CS6 (Adobe Systems Inc., San Jose, CA, USA). Criteria for identifying the structures of mitochondria, autophagic vacuoles and intercellular junctions were defined before the scoring was undertaken (Fig. 1). The characteristic double-membrane enclosed structures containing cristae or folds were scored as mitochondria (m, Fig. 1A). Several types of intercellular junctions (ij, Figs. 1C,1D) were found to be present in HCC70 cells. They were identified by the presence of electron-dense areas connecting the membranes. The structures enclosed by double membranes and containing unwanted or damaged cellular organelles, and partially digested material were considered as autophagic vacuoles (av, Fig. 1B) (Tu et al., 2011; Hale et al., 2013; Galluzzi & Kroemer, 2015).

Numbers for mitochondria, autophagic vacuoles and intercellular junctions were normalized to tissue area values that were measured using ImageJ software (Schneider, Rasband & Eliceiri, 2012). Briefly, the tissue area (µm^2^) of a TEM image was determined using the free-hand drawing tool. The counts for the number of structures were then divided by the tissue area of an image to calculate structures per unit area. Values were reported as number of structures per 100 µm^2^ of tissue area (File S1).

### Statistical analysis

The statistical analysis was carried out using Microsoft Excel 2011 for Macintosh (File S1). The counts obtained from six images of each block were grouped and means and totals were computed. A one-way Analysis of Variance (ANOVA) test was used to determine the statistical significance of the data collected from TEM images of monolayer and hydrogel cultures.

### Figure preparation

Phase contrast, confocal and TEM images were captured in TIFF format and saved at 300 dpi. Minimal image processing was carried out using ‘levels’ sliding bar, before labeling and composing figures using Adobe Photoshop CS6 (Adobe Systems Inc., San Jose, CA, USA). Microsoft Excel 2011 software for Macintosh was used for the preparation of graphs.

### Responsible conduct

The human breast cancer cell line HCC70 (ATCC^®^ CRL2315™; Lot #58033362; RRID: CVCL_1270) was obtained from the American Type Culture Collection (ATCC, Manassas, VA, USA) and cultured in the laboratory according to the vendor’s protocol. The use of the HCC70 cell line in a Biosafety Level 2 tissue culture facility was approved by the New Mexico State University Institutional Biosafety Committee (#1401SE2F0103). HCC70 is a commercial, publicly available cell line established using a mammary gland tissue from primary ductal carcinoma of a female donor, age 49 years, as listed on the vendor’s website (ATCC, Manassas, VA, USA). Consequently, HCC70 meets the exemption criteria for review by the Institutional Review Board under the Code of Federal Regulations 45 CFR 46.101(b)(4) (“Human Specimens, Cell Lines or Data — Research Involving Human Subjects,” 2016). The vendor has confirmed that the cells are of epithelial origin and are negative for three surface receptors: ER, PR and HER2.

## Results

### Phase contrast and confocal microscopy of monolayer and hydrogel cultures

**Figure 2:**
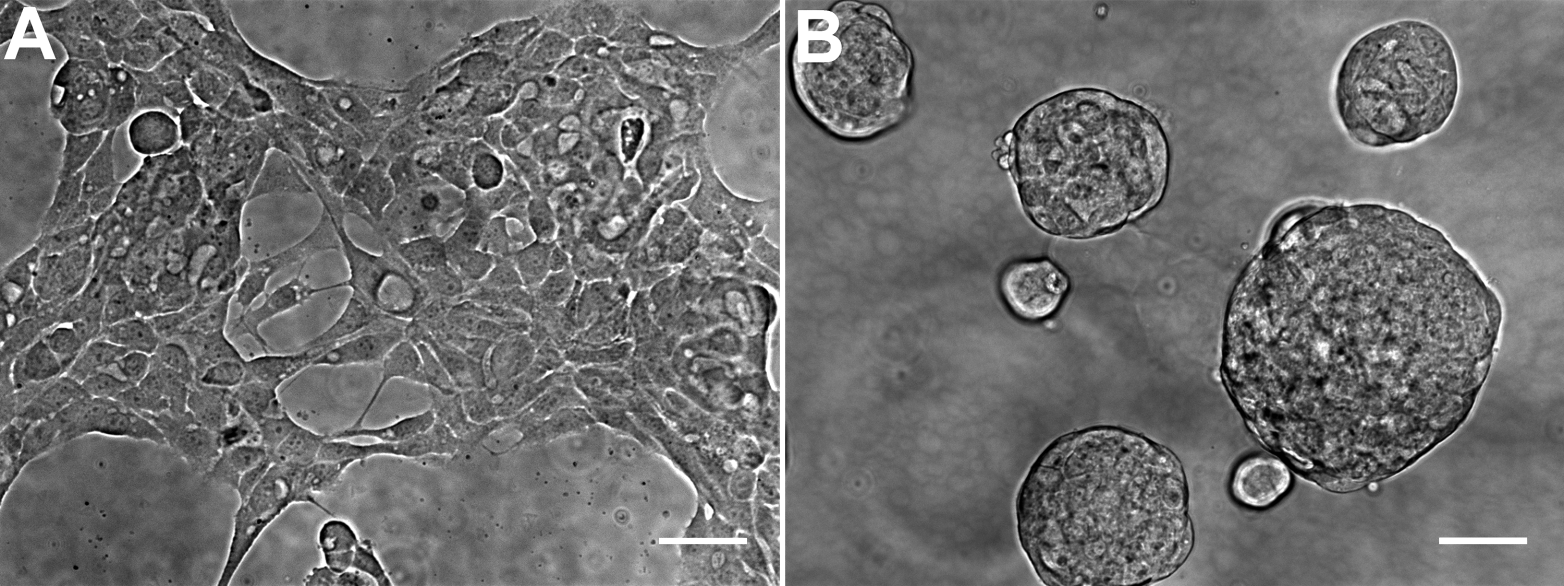
Phase contrast microscopy of HCC70 cells cultured in monolayer and hydrogel environments. (A) HCC70 cells cultured on rigid substratum showed the characteristic morphology described by the vendor (ATCC; Manassas, VA). Cells showed horizontal spreading, and close association with neighboring cells. (B) HCC70 cells formed spheroidal aggregates when cultured in the hydrogel. The data are representative of two independent experiments. Scale bar = 50 µm.

The general morphology of HCC70 cells was examined using phase contrast microscopy (Fig. 2). The monolayer and matrix cultures demonstrated strikingly different morphologies. Monolayer cells formed a single layer on the rigid substrate, spread horizontally, and appeared to contact neighboring cells (Fig. 2A). In contrast, HCC70 cells cultured with the basement membrane matrix Geltrex™ formed compact aggregates and spheroidal structures (Fig. 2B).

**Figure 3:**
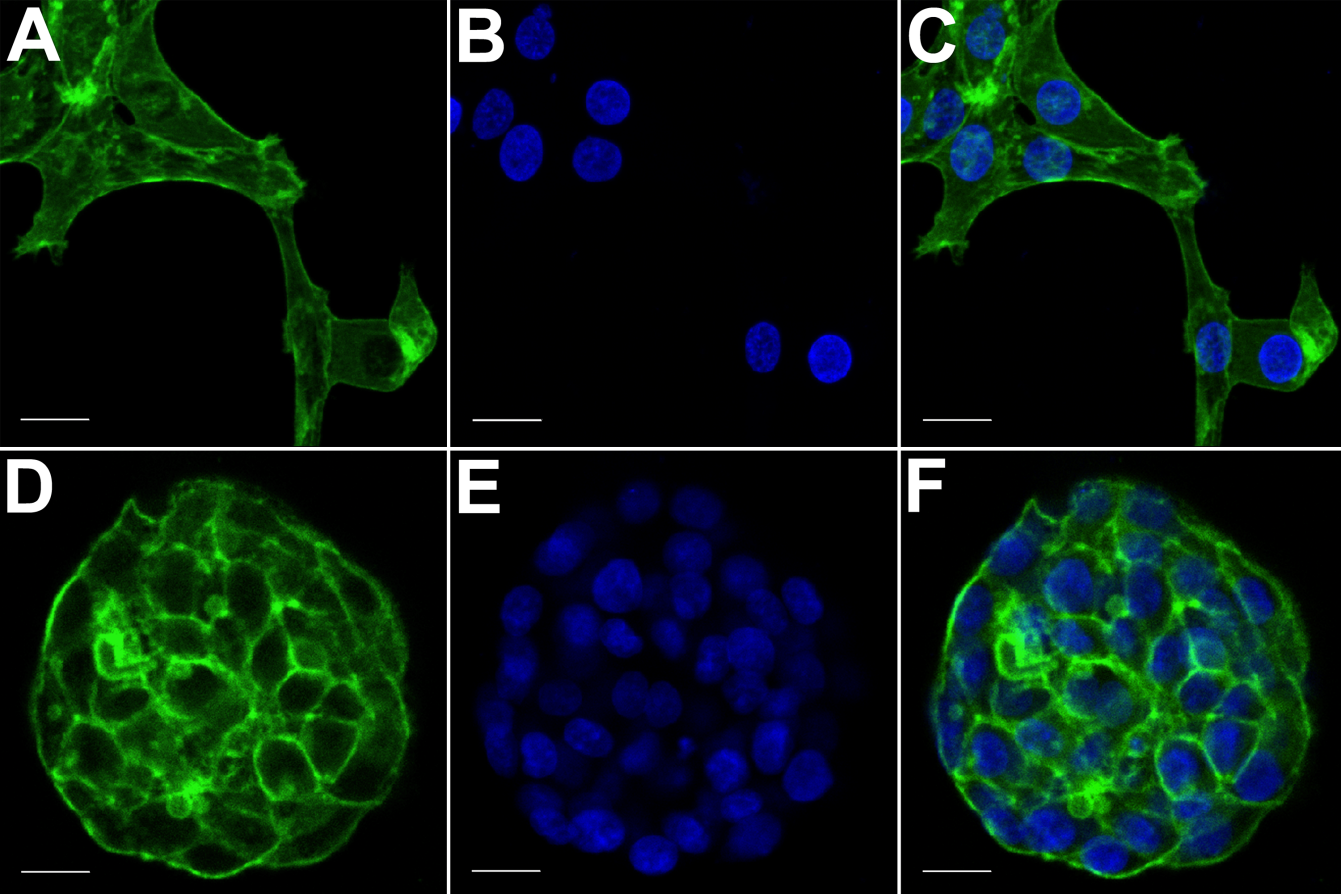
Confocal microscopy of HCC70 cells cultured in monolayer and hydrogel environments. The spatial arrangements of actin-based structures and cell nuclei in monolayer and hydrogel cultures of HCC70 cells were visualized using confocal microscopy by labeling with fluorescent probes—Alexa Fluor^®^488 Phalloidin (green) for F-actin and Hoechst 33342 (blue) for DNA. (A–C) During confocal projections of labeled cells, monolayer cells appeared flat. The cells also appeared to be situated far from each other. (D–F) In hydrogel environment, cells formed spheroids and the cells appeared to adhere closely to one another. Scale bar = 10 µm.

The morphology of monolayer and matrix cultures was further analyzed using laser confocal microscopy to study the spatial arrangement of cells and localize actin filaments and DNA. The two groups of cells were stained using Alexa Fluor^®^ 488 Phalloidin dye specific for F-actin and Hoechst 33342, a DNA-specific probe (Fig. 3). Confocal images of HCC70 cells in monolayer cultures indicated that cells were flattened and adhered to the rigid glass substratum, while cells cultured in the hydrogel formed well-rounded, multilayered structures. We noted that inter-nuclear distances between monolayer cells (Figs. 3A–3C) were higher than those in matrix-organized cells (Figs. 3D–3F).

### Ultra-structural analysis of monolayer cultures

**Figure 4:**
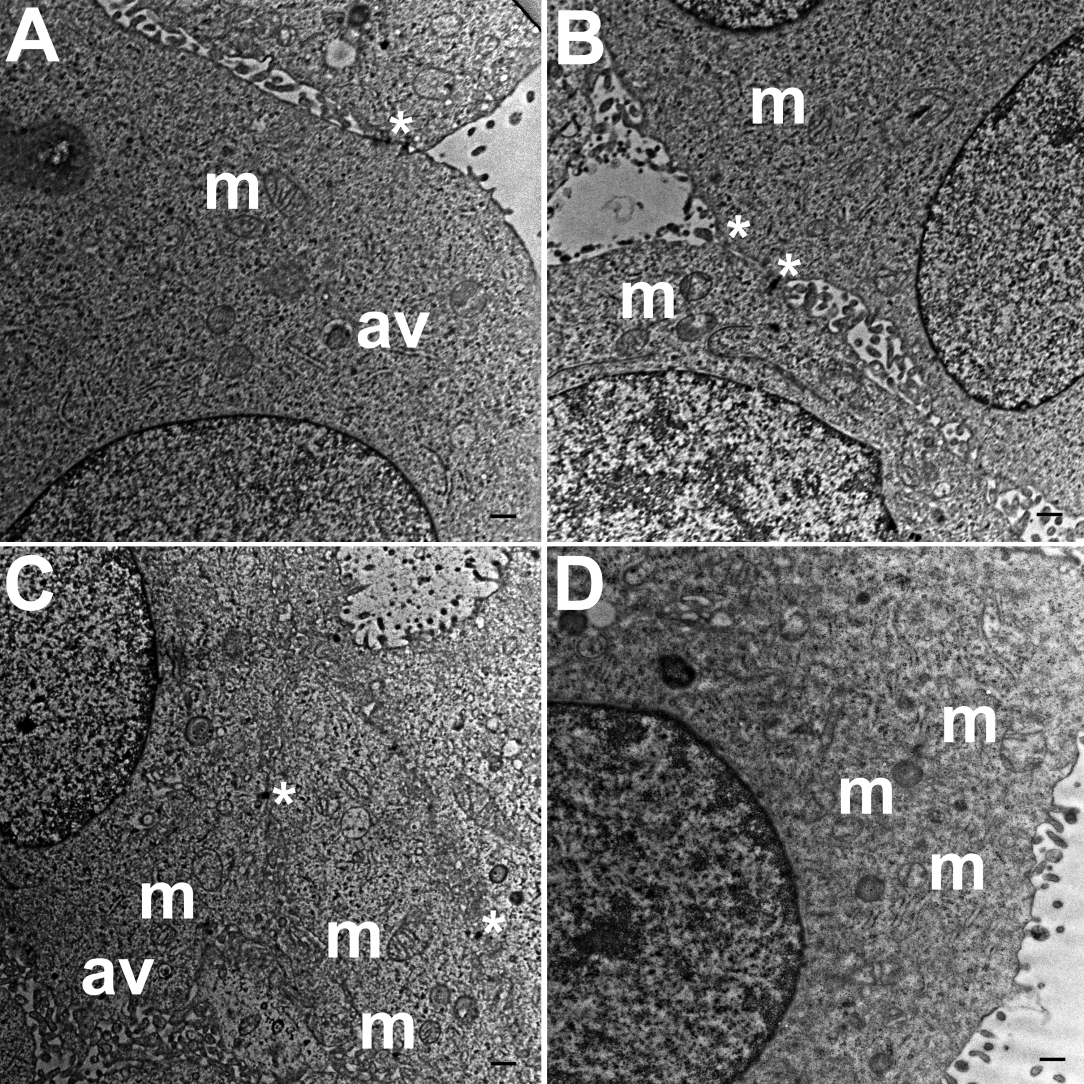
Exemplar TEM images of HCC70 cells cultured in monolayer environment. Cells cultured on rigid substratum consisted of mitochondria (A–D,‘m’) and autophagic vacuoles (A, C,‘av’). The cells were adhered to the neighboring cells with the help of intercellular junctions (A–C,‘*’). The data are representative of two independent experiments (A, B: Experiment 1; C, D: Experiment 2). Scale bar = 500 nm.

TEM images of HCC70 cells cultured in a monolayer environment showed that cells adhered to adjacent cells with the help of intercellular junctions (Figs. 4A–4C, *). A few mitochondria (Figs. 4A–4D, m) and autophagic vacuoles (Figs. 4A, 4C, av) were observed inside the cells. The mitochondria had a variety of shapes such as round, oval, and cylindrical. The sum of individual counts for mitochondria, autophagic vacuoles and intercellular junctions is shown in Table 1 together with the sampled area.

**Table 1:** Quantitative analysis of TEM images captured from monolayer and hydrogel cultures of HCC70 cells. TEM images were captured from four blocks for each condition—monolayer and hydrogel. The number of mitochondria, autophagic vacuoles and intercellular junctions were counted from six images obtained from each block. The numbers shown in the table represent the sum of counts obtained from six images.

Culture Type	Analysis Area (µm^2^)	Mitochondria	Autophagic Vacuoles	Intercellular Junctions
Monolayer_Block 1	783.6	42	37	38
Monolayer_Block 2	780.3	32	14	40
Monolayer_Block 3	771.0	42	21	36
Monolayer_Block 4	788.7	25	29	24
Hydrogel_Block 1	785.9	88	47	57
Hydrogel_Block 2	800.4	80	60	61
Hydrogel_Block 3	800.2	80	70	71
Hydrogel_Block 4	776.7	71	45	65

### Ultra-structural analysis of hydrogel cultures

**Figure 5:**
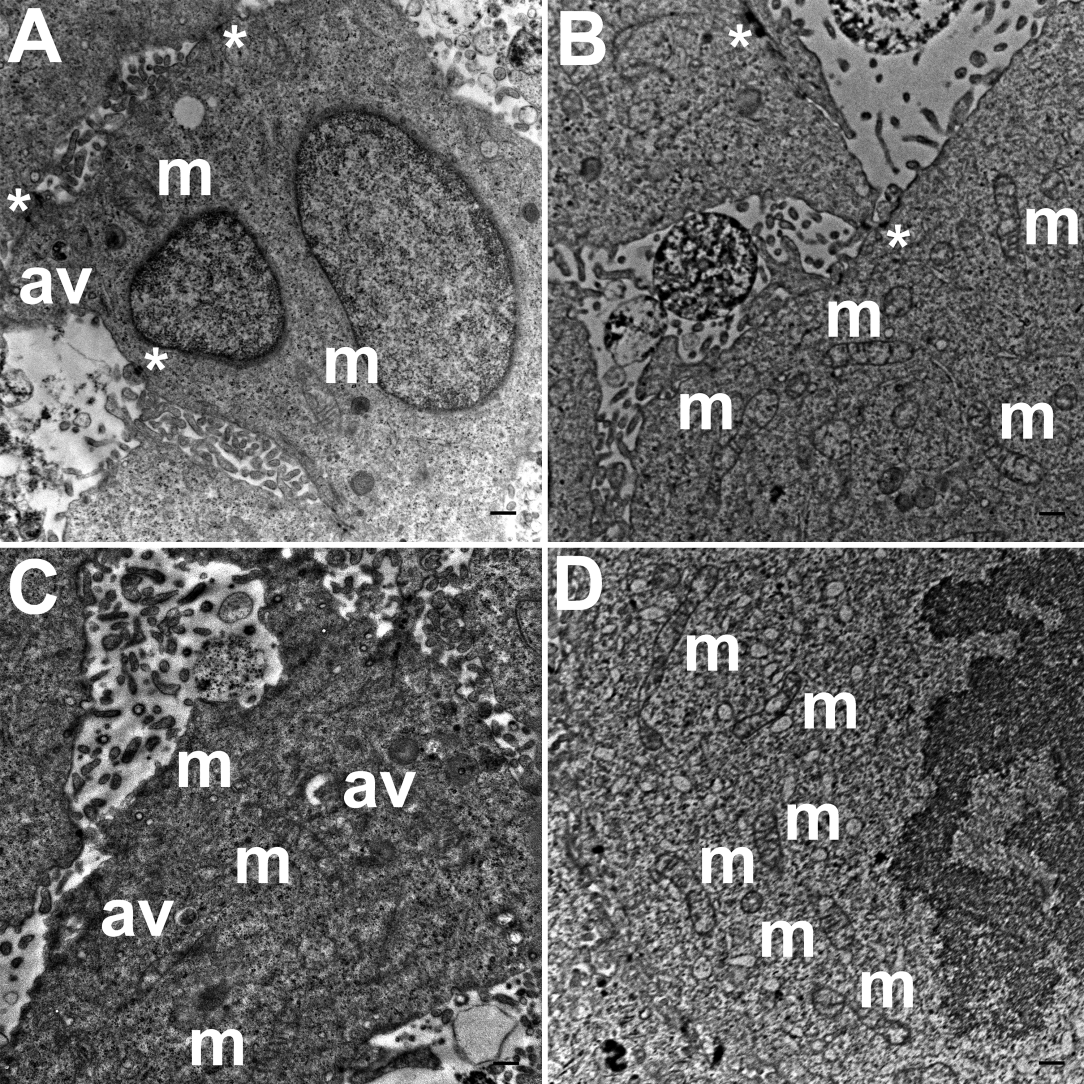
Exemplar TEM images of HCC70 cells cultured in hydrogel. HCC70 cells grew adjacent to each other in hydrogel and comprised several mitochondria (A–D,‘m’), autophagic vacuoles (A, C,‘av’) and intercellular junctions (A, B,‘*’). The data are representative of two independent experiments (A, B: Experiment 1; C, D: Experiment 2). Scale bar = 500 nm.

TEM images of hydrogel cultures of HCC70 cells showed that cells formed multiple intercellular junctions with neighboring cells (Figs. 5A, 5B, *). Cells in hydrogels comprised autophagic vacuoles (Figs. 5A, 5C, av), as well as mitochondria with a variety of shapes ranging from round to oval to cylindrical (Figs. 5A–5D, m). The sampled area, the sum of individual counts for mitochondria, autophagic vacuoles and intercellular junctions are shown in Table 1.

**Figure 6:**
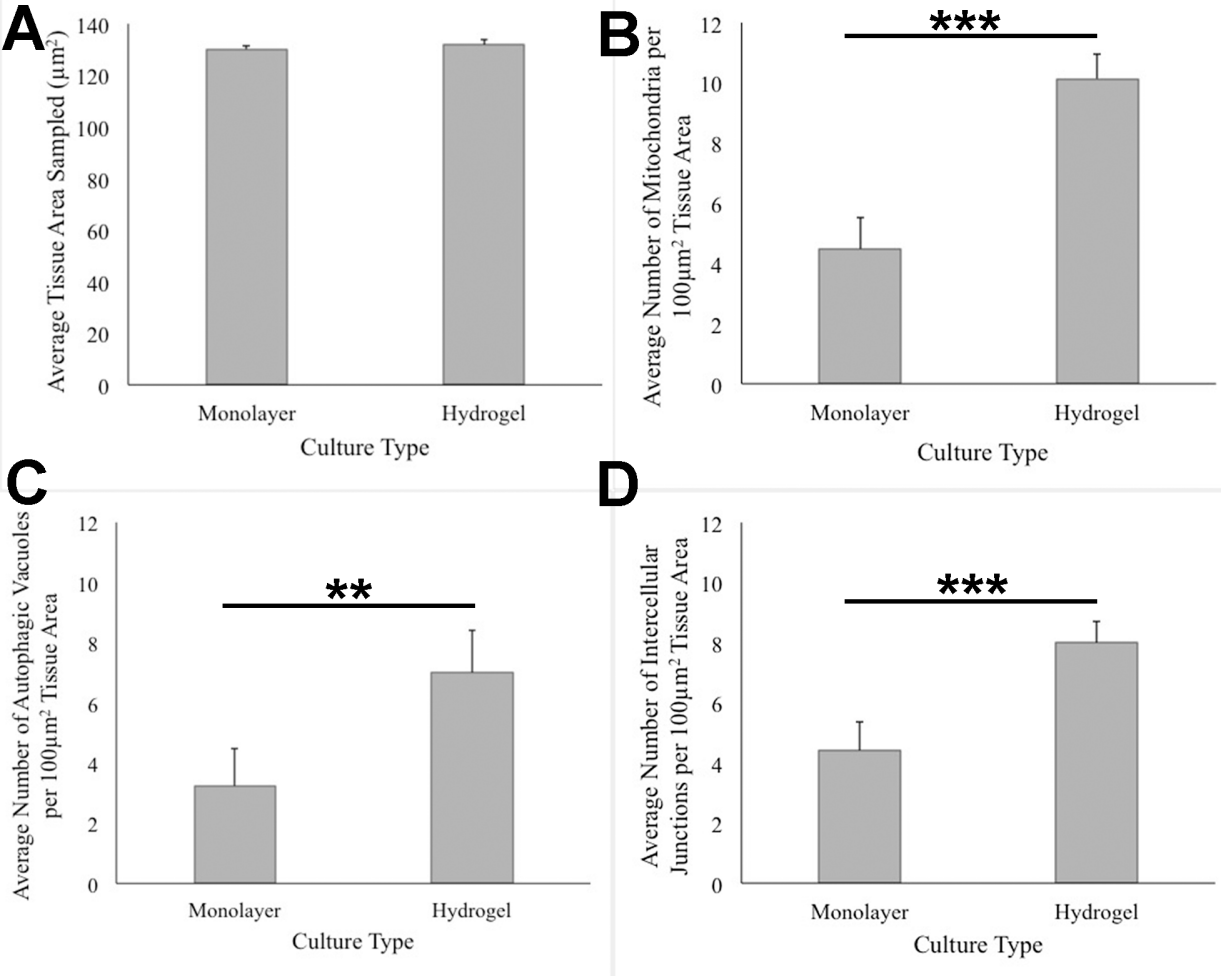
Quantitative analysis of tissue area, mitochondria, autophagic vacuoles, and intercellular junctions present in TEM images from monolayer and hydrogel cultures of HCC70. The tissue area (A), the number of mitochondria (B), number of autophagic vacuoles (C), and the number of intercellular junctions were counted in images from resin blocks prepared from monolayer and hydrogel cultures (6 images/block; 4 blocks from each condition). The histograms compare the mean ± S.D. computed from the 4 means for each condition using values for the parameters that were normalized to 100 µm^2^ tissue area. Statistical significance values were estimated with the Analysis of Variance (ANOVA) test. ** *P* ≤ 0.01, *** *P* ≤ 0.001.

ANOVA analysis of ultrastructure data from the two growth conditions showed that HCC70 cells cultured in hydrogel comprised a higher number of mitochondria (*Pvalue* < 0.001), autophagic vacuoles (*Pvalue* < 0.01) and intercellular junctions (*Pvalue* < 0.001) than those present in monolayer cells (Figs. 6B–6D). The tissue area used for quantitative analysis was equivalent (*Pvalue* > 0.05) for both culture conditions (Fig. 6A).

## Discussion

Marked morphological differences were observed between HCC70 monolayer and hydrogel cultures with light microscopy, especially in the types of associations cells formed with one another. Phase contrast (Fig. 2) and confocal (Fig. 3) images captured from cells cultured in monolayer environments showed the characteristic morphology specified by the vendor (ATCC, Manassas, VA, USA). Cells were phase-dark, indicating a flattened morphology, and spread horizontally on the surface of a glass slide. In contrast, the cells cultured in hydrogel grew in stacked layers and formed spheroidal masses that resembled ‘mammospheres’ (Manuel Iglesias et al., 2013) and multicellular tumor spheroids (Nath & Devi, 2016). When cells labeled with fluorescent probes for F-actin and nuclei were imaged with confocal microscopy, we found that the monolayer cells were situated far from one other as compared to HCC70 cells forming compact spheroids in hydrogel (Fig. 3).

Our results are consistent with previous studies conducted using breast cancer cell lines where it has been shown that cells exhibit distinct morphologies in monolayer versus hydrogel culture environments (Kenny et al., 2007; Vidi, Bissell & Lelièvre, 2013). HCC70 cells that we cultured in the hydrogel scaffold Geltrex™ formed spheroids that resembled the “Mass”-like aggregates previously reported for this cell line when cultured in a laminin-rich extracellular matrix hydrogel (Kenny et al., 2007). It is well established that the aforementioned morphological changes elicited by hydrogels are not restricted to cells of neoplastic origin. Non-cancerous mouse and human cell lines derived from normal cardiac and mammary epithelial tissue also have been shown to be morphologically distinct when cultured in hydrogel versus monolayer conditions, underscoring the global effect of culture substratum and environment on cell differentiation, regardless of tissue provenance (Emerman, Burwen & Pitelka, 1979; Streuli, Bailey & Bissell, 1991; Underwood et al., 2006; Liao et al., 2007; Pontes Soares et al., 2012). Research conducted using normal mammary gland epithelial cells and human breast cancer cells suggests that morphological changes visible in hydrogels may be due to regulation of cell growth and migration by matrix stiffness, and that cell viability decreases with increase in elasticity of hydrogels, with higher cell proliferation rate in soft hydrogels (Bissell & Barcellos-Hoff, 1987; Cavo et al., 2016). These intriguing observations raise questions about the mechanisms that promote HCC70 cell division in Geltrex™, a topic for future investigations using proliferation markers such as Ki67 (Pan et al., 2017).

TEM images of monolayer and hydrogel cultures showed that cells comprised typical subcellular organelles and structures (Figs. 4, 5). However, quantitative analysis of TEM images from HCC70 cultures uncovered a higher number of mitochondria, intercellular junctions and autophagic vacuoles in cells grown in hydrogel environments. It is notable that organelle dysfunction and heterogeneity are emerging as features of disease states such as neurodegeneration and cancer (Marshall, 2007; Mukherji & O’Shea, 2014; Chang & Marshall, 2017). For example, Ren et al. (1990) found that the presence of a higher number of mitochondria in rat mammary tumor tissues was representative of highly metastatic tumors, suggesting that the TNBC hydrogel cultures may mimic the tumor state more closely than monolayer systems with regard to mitochondrial role in cancer progression (Ren et al., 1990). Similarly, the increase in intercellular junctions may be attributed to a greater requirement for robust cell–cell adhesion when spheroid masses are formed as compared with more restricted cell–cell contact at flattened edges during monolayer growth (Kenny et al., 2007). Autophagy also has been shown to play a key role in the progression of many diseases such as cancer, neurodegenerative disorders and infectious diseases (Galluzzi & Kroemer, 2015). For example, in cancer, autophagy can act as a tumor suppressor during the initial stages of tumor progression, but may promote tumor cell survival in later stages (Tu et al., 2011; Mah & Ryan, 2012; Kaminskyy et al., 2012; Huang et al., 2014; Morgan et al., 2014; Galluzzi et al., 2015). Lefort et al. (2014) have shown that autophagy-linked genes are upregulated in TNBC cell lines, including the HCC70 cell line that was used in the current study, cultured in a laminin-rich extracellular matrix (Lefort et al., 2014). Moreover, TNBC cell lines, including HCC70, appear to respond to the suppression of the NEET family proteins, mitoNEET (mNT) and Nutrient-deprivation autophagy factor-1 (NAF-1), with impaired ultrastructural mitochondrial morphology and autophagosome accumulation (Sohn et al., 2013). Research undertaken with melanoma cell lines suggests that higher autophagy is indicative of a more aggressive tumor state, resistance to treatment, and poor patient survival (Ma et al., 2011). These findings point to a critical role for autophagy in regulating cell survival and imparting drug-resistance to cells (Zou et al., 2012). The greater incidence of autophagic vacuoles in cells cultured in hydrogels may signify that the matrix environment is stimulating autophagy pathways in the HCC70 cells.

## Conclusion

In this study, we developed protocols for ultrastructural analysis of HCC70 cells grown in hydrogel and monolayer culture conditions. These methods may be useful for other researchers who seek to capture and analyze TEM image data from hydrogel cultures of cancer and normal cell lines. Our results demonstrated that HCC70 cells grown in a hydrogel microenvironment modified their morphology, cell–cell organization, and organelle abundance in comparison with monolayer cultures in a manner consistent with features of more aggressive tumors (Ren et al., 1990; Ma et al., 2011; Zou et al., 2012; Sohn et al., 2013). Future experiments aim to uncover the molecular mechanisms underlying the observed changes between monolayer and hydrogel cultures using molecular markers and functional assays specific for mitochondria, autophagic vacuoles, and intercellular junctions.

##  Supplemental Information

10.7717/peerj.4340/supp-1 Supplemental Information 1 Organelle counts for monolayer and hydrogel cultures and statistical analysis Counts for the number of mitochondria, autophagic vacuoles and intercellular junctions were determined from TEM images of HCC70 cells grown in monolayer and hydrogel cultures. The data are shown as number of structures/100 µm^2^ of tissue area. The statistical analysis was conducted using ANOVA test.

10.7717/peerj.4340/supp-2 Supplemental Information 2 Cell line IRB exemption Documentation for cell line exemption status (NMSU and NIH).

## List of Abbreviations

2D: two-dimensional
3D: three-dimensional
ANOVA: one-way Analysis of Variance
BSA: bovine serum albumin
ddH_2_O: double distilled water
FBS: fetal bovine serum
PBS: phosphate buffered saline
TEM: transmission electron microscopy
TNBC: triple negative breast cancer
UV: ultraviolet

## Additional Information and Declarations

### Data Availability

The following information was supplied regarding data availability: The raw data is provided in the Supplemental File.
