# Supplementary material for: Morphometric analysis of a triple negative breast cancer cell line in hydrogel and monolayer culture environments
Source: PeerJ. 2018 Feb 16;6:e4340. doi: 10.7717/peerj.4340 (PMC5817938; doi:10.7717/peerj.4340)
Supplement: Supplemental Information 2 — Documentation for cell line exemption status (NMSU and NIH). [file peerj-06-4340-s002.pdf]

As stated in the manuscript and in the submission fields, the CCF-STTG1 Cell line is purchased from ATCC, the American Type Culture Collection. https://www.atcc.org/products/all/CRL-1718.aspx#generalinformation

The NMSU policy is posted on the web page: http://compliance.research.nmsu.edu/IRBexempt

> *Item 4 applies: "4. Existing Data or Specimens*
> *Research involving the collection or study of existing data documents, records, pathological specimens, or diagnostic specimens, if these sources are publicly available or if the information is recorded by the investigator in such a manner that subjects cannot be identified, directly or through identifiers linked to the subjects. "*

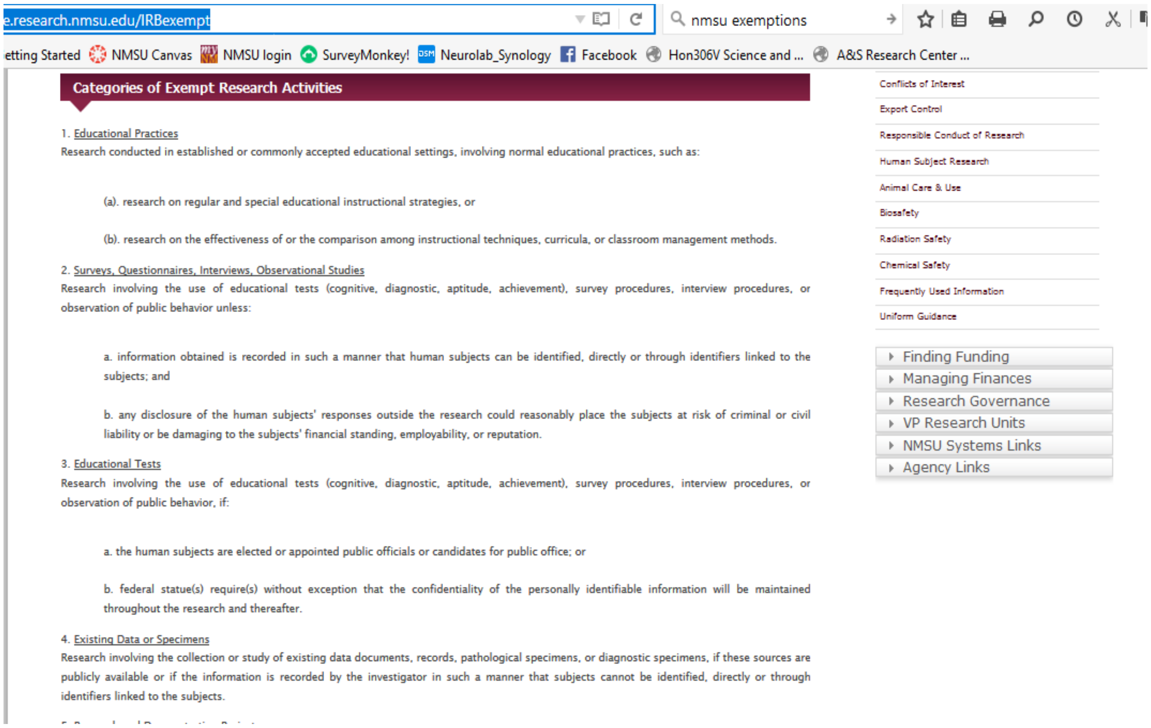

e.research.nmsu.edu/IRBexempt

etting Started NMSU Canvas NMSU login SurveyMonkey! Neurolab_Synology Facebook Hon306V Science and ... A&S Research Center ...

Categories of Exempt Research Activities

1. Educational Practices
Research conducted in established or commonly accepted educational settings, involving normal educational practices, such as:

(a). research on regular and special educational instructional strategies, or

(b). research on the effectiveness of or the comparison among instructional techniques, curricula, or classroom management methods.

2. Surveys, Questionnaires, Interviews, Observational Studies
Research involving the use of educational tests (cognitive, diagnostic, aptitude, achievement), survey procedures, interview procedures, or observation of public behavior unless:

a. information obtained is recorded in such a manner that human subjects can be identified, directly or through identifiers linked to the subjects; and

b. any disclosure of the human subjects' responses outside the research could reasonably place the subjects at risk of criminal or civil liability or be damaging to the subjects' financial standing, employability, or reputation.

3. Educational Tests
Research involving the use of educational tests (cognitive, diagnostic, aptitude, achievement), survey procedures, interview procedures, or observation of public behavior, if:

a. the human subjects are elected or appointed public officials or candidates for public office; or

b. federal statue(s) require(s) without exception that the confidentiality of the personally identifiable information will be maintained throughout the research and thereafter.

4. Existing Data or Specimens
Research involving the collection or study of existing data documents, records, pathological specimens, or diagnostic specimens, if these sources are publicly available or if the information is recorded by the investigator in such a manner that subjects cannot be identified, directly or through identifiers linked to the subjects.

Conflicts of Interest
Export Control
Responsible Conduct of Research
Human Subject Research
Animal Care & Use
Biosafety
Radiation Safety
Chemical Safety
Frequently Used Information
Uniform Guidance

Finding Funding
Managing Finances
Research Governance
VP Research Units
NMSU Systems Links
Agency Links

NIH states the following: https://humansubjects.nih.gov/human-specimens-cell-lines-data

> ***"Research that proposes the use of human cell lines available from the American Type Culture Collection or a similar repository is not considered human subjects research because the cells are publicly available and all of the information known about the cell lines (perhaps, including the donor) is also publicly available."***

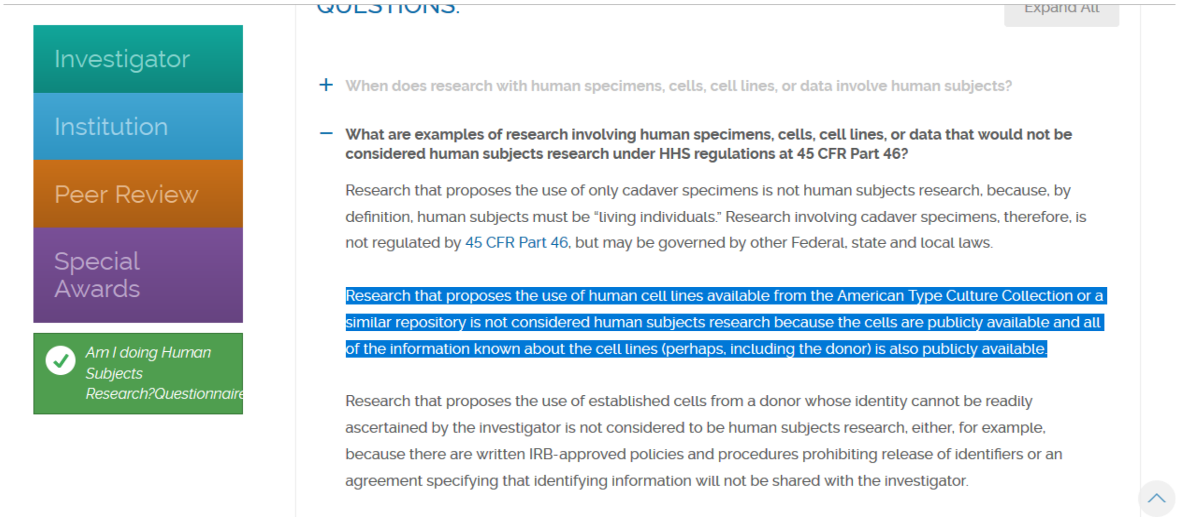

Investigator
Institution
Peer Review
Special Awards
Am I doing Human Subjects Research?Questionnaire

Expand All

\+ When does research with human specimens, cells, cell lines, or data involve human subjects?

− **What are examples of research involving human specimens, cells, cell lines, or data that would not be considered human subjects research under HHS regulations at 45 CFR Part 46?**

Research that proposes the use of only cadaver specimens is not human subjects research, because, by definition, human subjects must be "living individuals." Research involving cadaver specimens, therefore, is not regulated by 45 CFR Part 46, but may be governed by other Federal, state and local laws.

Research that proposes the use of human cell lines available from the American Type Culture Collection or a similar repository is not considered human subjects research because the cells are publicly available and all of the information known about the cell lines (perhaps, including the donor) is also publicly available.

Research that proposes the use of established cells from a donor whose identity cannot be readily ascertained by the investigator is not considered to be human subjects research, either, for example, because there are written IRB-approved policies and procedures prohibiting release of identifiers or an agreement specifying that identifying information will not be shared with the investigator.
